# Efficacy and safety of acupuncture for early postpartum stress urinary incontinence: A protocol for a pilot randomized controlled trial

**DOI:** 10.1371/journal.pone.0324384

**Published:** 2025-05-27

**Authors:** Yi Yang, Meng Xu, Qingguo Liu, Xiaoqing Ding, Xi Wang, Dongfeng Wang, Qisheng Sun, Xiaowei Shi, Xiuping Zhang, Dong Liu, Shufeng Shi

**Affiliations:** 1 School of Acupuncture-Moxibustion and Tuina, Beijing University of Chinese Medicine, Beijing, China; 2 Department of Tuina, Beijing University of Chinese Medicine Third Affiliated Hospital, Beijing, China; 3 St. Petersburg Chinese Medicine Center, Beijing University of Chinese Medicine, St. Petersburg, Russia; 4 Heilongjiang University of Chinese Medicine, Harbin, China; 5 Department of Tuina, Miyun District Traditional Chinese Medicine Hospital, Beijing, China; 6 Department of Gynaecology, Beijing University of Chinese Medicine Third Affiliated Hospital, Beijing, China; 7 Department of Urology, Beijing University of Chinese Medicine Third Affiliated Hospital, Beijing, China; China Medical University, TAIWAN

## Abstract

**Objective:**

Postpartum stress urinary incontinence (PSUI) is a common condition among women after childbirth. While acupuncture is a common clinical treatment for PSUI, high-quality clinical evidence supporting its effectiveness is currently lacking. This study aims to preliminarily evaluate the efficacy and safety of acupuncture for early postpartum stress urinary incontinence, providing reference data for sample size calculation and protocol feasibility in formal trials.

**Design and methods:**

This is a randomized controlled trial. Seventy-two PSUI patients between 42 days and 1 year postpartum will be randomly assigned in a 1:1 ratio to either the acupuncture group (n = 36) or the sham acupuncture group (n = 36). Both groups will receive acupuncture treatments three times per week for two weeks. Both groups will receive identical education regarding pelvic floor muscle training. The primary outcomes are changes in urine leakage volume measured by 1-hour pad test at week 2 of treatment and week 6 of follow-up compared to baseline (week -1), and the proportion of patients achieving at least 50% reduction in urine leakage volume at week 2 compared to baseline. Secondary outcomes include 72-hour bladder diary, The International Consultation on Incontinence Questionnaire-Short Form (ICIQ-SF), Incontinence Quality of Life questionnaire (I-QOL), and pelvic floor muscle strength assessment.

**Discussion:**

The results of this study will provide preliminary evidence on the efficacy and safety of acupuncture for PSUI, offering reference data for sample size calculation and protocol feasibility in formal trials, ultimately providing more treatment options for PSUI patients.

**Trial registration:**

ITMCTR2024000290.

## Introduction

Stress urinary incontinence (SUI) is a common condition in women characterized by involuntary urine leakage during sudden increases in abdominal pressure, such as coughing, laughing, sneezing, or lifting heavy objects, despite having no urinary symptoms under normal circumstances [1]. As a crucial public health issue globally, urinary incontinence (UI) affects the physical and mental well-being of approximately 45% of adult women, with stress urinary incontinence (SUI) being the predominant subtype, accounting for over half of UI cases. In the United States, the prevalence of UI from 2005 to 2016 was 53%, with 26% of women suffering from SUI [2]. In mainland China, the prevalence of SUI among adult women is 24.5% [3].SUI patients often experience incontinence in public settings, leading to body odor issues that can trigger anxiety, fear, and depression in social and work situations. Although SUI is not life-threatening, it significantly impacts patients’ daily lives, work, and social activities. Based on these considerations, World Health Organization (WHO) has identified SUI as a social issue affecting global women’s quality of life and mental health [4].

Postpartum stress urinary incontinence (PSUI) is a common condition in women after pregnancy and childbirth. Studies show the incidence of postpartum urinary incontinence (PPUI) is around 26% [5], with stress UI (54%) being the most prevalent type, affecting first-time and experienced mothers similarly [6].

For mild to moderate PSUI, conservative treatments are typically used, while severe cases are often recommended for surgery. International guidelines recommend lifestyle modifications, behavioral therapy, pelvic floor muscle training (PFMT), and functional electrical stimulation as standard conservative therapies for mild to moderate female stress UI. PFMT is the first-line conservative treatment, with 30–60% efficacy, but requires at least 3 months of adherence to achieve maximum benefits [7]. However, many patients struggle to adhere to PFMT. Biofeedback combined with electrical stimulation is a common clinical approach, but its long-term efficacy is inconsistent, and the treatment is costly for patients [8]. Existing PSUI treatments have limitations, highlighting the need for alternative or supplementary therapies.

Traditional Chinese medicine offers various SUI treatments, with acupuncture showing the most promising results. A 504-patient RCT found electroacupuncture significantly reduced urine leakage and improved quality of life in middle-aged/elderly women, with clear clinical efficacy and safety [9]. PSUI differs from general SUI, as PSUI is caused by pelvic floor and supporting structure damage from pregnancy/childbirth, while SUI often results from pelvic organ prolapse or muscle weakness. Therefore, the symptoms and treatments for PSUI and SUI are not entirely identical. Previous research has explored acupuncture for PSUI, but evidence on its efficacy is limited [10–12]. High-quality randomized controlled trials are needed to thoroughly evaluate the effectiveness and safety of acupuncture for PSUI treatment. Positive findings could provide a new clinical approach for PSUI while bolstering Traditional Chinese Medicine’s application in modern medicine.

This pilot trial aims to provide evidence that acupuncture has therapeutic effects on PSUI beyond placebo. Prior studies lack sham-controlled acupuncture as a comparator for PSUI [10–12]. To our knowledge, this will be the first acupuncture trial using non-acupoint, shallow, and non-deqi needling as a sham control to rigorously evaluate acupuncture’s efficacy for PSUI. If the true acupuncture group shows superior results, it would preliminarily confirm acupuncture’s therapeutic benefits and inform future formal trials.

The primary objective of this trial is to verify the feasibility of the protocol, defined as meeting the following criteria: (1) dropout rate ≤20%; (2) compliance rate ≥80%; (3) data completeness ≥90%; (4) adverse event rate <5%, with no intervention-related serious adverse events. The secondary objective is to collect preliminary parameters for sample size calculation, including: (1) mean between-group difference in the 1-hour pad test; (2) standard deviation of outcome measurements; (3) estimated effect size (Cohen’s d).

Two hypotheses are proposed: (1) Acupuncture can reduce urine leakage volume in patients with PSUI; (2) Acupuncture is a safe intervention for treating PSUI.

## Methods

### Study design

**Fig 1:**
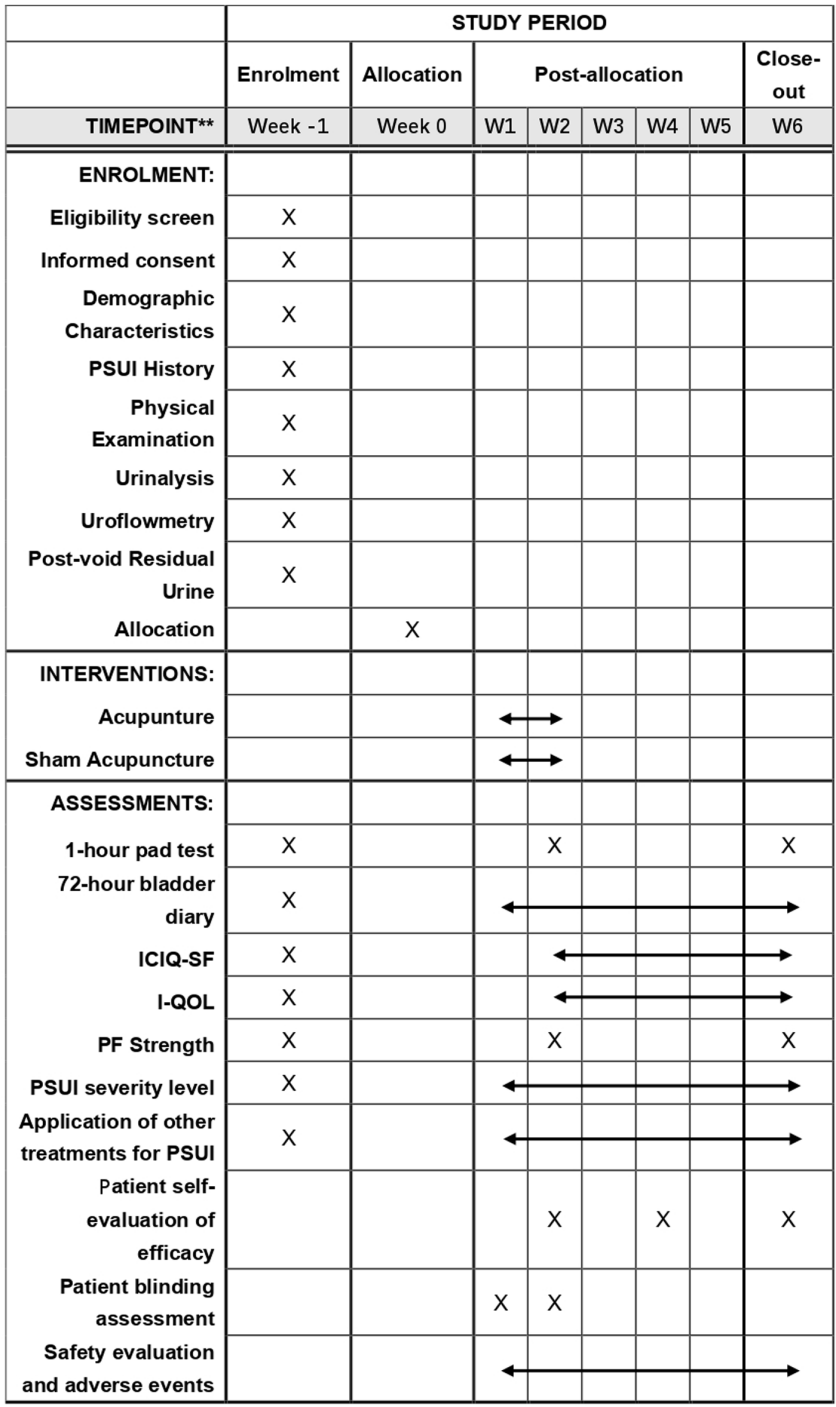
SPIRIT schedule. Enrollment, Intervention, and Assessment Schedule. PSUI, Postpartum Stress Urinary Incontinence; ICIQ-SF, The International Consultation on Incontinence Questionnaire-Short Form; I-QOL, Incontinence Quality of Life; PF Strength, Pelvic Floor Strength Assessment.

**Fig 2:**
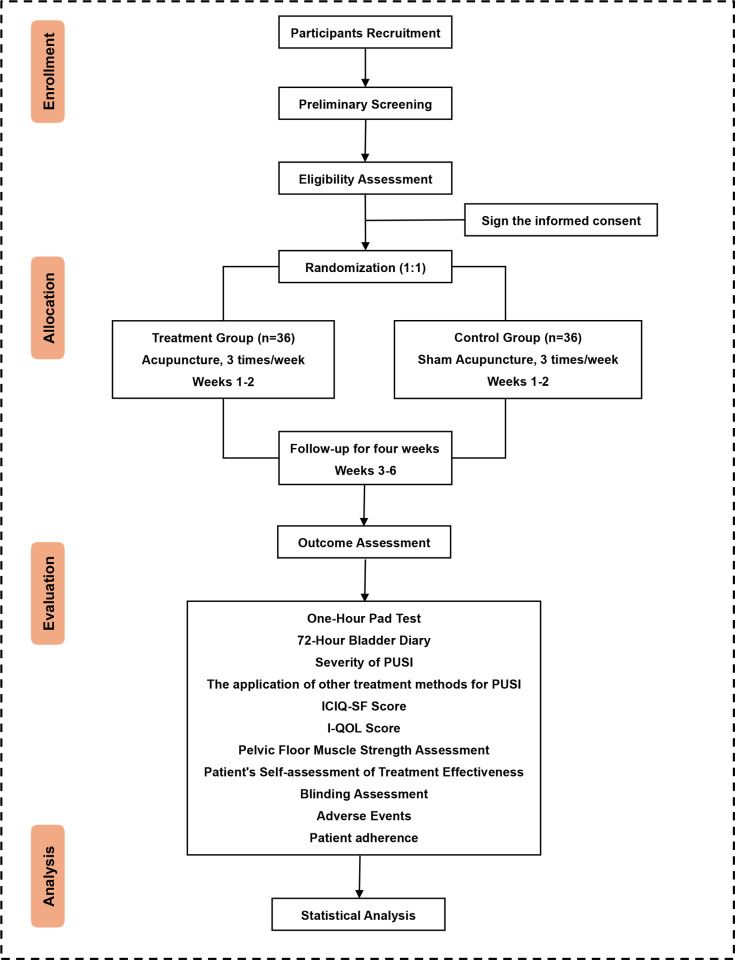
The flow chart of this trial.

This is a pragmatic, single-center, two-arm, randomized controlled trial conducted in China. A total of 72 postpartum women with stress urinary incontinence will be randomly allocated in a 1:1 ratio to an acupuncture group and a sham acupuncture group. The trial duration is six weeks, including a two-week treatment period and a four-week follow-up. The study protocol has been approved by the Ethics Committee of Beijing University of Chinese Medicine Third Affiliated Hospital (Approval No. BZYSY-2024KYKTPJ-23) and registered on the International Traditional Medicine Clinical Trial Registry platform (ITMCTR, [http://itmctr.ccebtcm.org.cn/]) with registration number ITMCTR2024000290 (Date: August 24, 2024, version 4.0). The trial design adheres to the Standards for Reporting Interventions in Clinical Trials of Acupuncture (STRICTA) [13] and the Standard Protocol Items: Recommendations for Interventional Trials (SPIRIT) guidelines [14]. Fig 1 illustrates the SPIRIT schedule, and S1 File shows the SPIRIT checklist. Fig 2 illustrates the flow chart of this trial.

### Study site

Recruitment, intervention and follow up will be conducted at Beijing University of Chinese Medicine Third Affiliated Hospital. Following randomization, patients will be allotted to the respective group. Recruitment will take place on outpatient departments, with intervention and follow up occurring in dedicated rooms within the hospitals.

### Participants

#### Inclusion criteria.

Patients will be eligible if they meet the following criteria:

(1) Aged between 20 and 40 years;
(2) Within 42 days to 1 year postpartum;
(3) Experience urinary leakage during activities such as coughing, sneezing, laughing, or physical exertion, which ceases when the activity stops, occurring ≥1 time in the past three months;
(4) Demonstrate an increase in pad weight of more than 1 g but less than 50 g during a 1-hour pad test;
(5) Diagnosed with mild to moderate SUI based on the Ingelman-Sundberg classification;
(6) Voluntarily agree to participate in the study and sign the informed consent form.

Diagnosis and Ingelman-Sundberg grading will be conducted by a urologist.

#### Exclusion criteria.

Patients will be ineligible if meet any of the following points:

(1) Multiple gestation pregnancy;
(2) Pre-pregnancy urinary incontinence symptoms;
(3) Urinary frequency and urgency symptoms;
(4) Symptoms of urinary tract infection;
(5) Incomplete lochia discharge postpartum or presence of intrauterine retention;
(6) Concurrent uterine or other gynecological diseases;
(7) History of severe pregnancy complications, including: macrosomia, prolonged second stage of labor, placenta previa, threatened preterm labor and gestational hypertension;
(8) Previous indications for cesarean section or contraindications to vaginal delivery;
(9) Previous urinary incontinence surgery or pelvic floor surgery;
(10) Pelvic organ prolapse greater than grade 2;
(11) Overactive bladder syndrome or urinary system tumors;
(12) Detrusor muscle weakness;
(13) Specific treatment for SUI within the past month or use of medications that may affect bladder function;
(14) Severe conditions including: cardiovascular disease, cerebral disease, hepatic disease, renal disease, psychiatric disorders, diabetes mellitus, multiple system atrophy, cauda equina injury and spinal cord injury;
(15) Presence of cardiac pacemaker, metal allergy, or severe needle phobia;
(16) Participation in other clinical trials within the past 3 months.

#### Shedding criteria.

Subjects who, despite providing informed consent form and passing the screening process to obtain a randomization number, are unable to complete the treatment regimen and observation period specified in the study protocol are considered shedding cases.

#### Management of shedding cases.

When a subject is shed, efforts should be made to contact them via home visits, scheduled follow-ups, telephone calls, or letters to determine the reasons for shedding. Relevant information should be recorded, including the date of the last acupuncture session and the evaluation items completed up to that point. All data pertaining to shedding cases should be carefully preserved, both for archival purposes and for inclusion in intention-to-treat (ITT) statistical analysis.

#### Termination criteria.

(1) At least one urologist or gynecologist must participate in this study. If a patient experiences severe adverse reactions that, in the physician’s judgment, necessitate the termination of their participation in the clinical trial, the case should be withdrawn.
(2) If the patient develops other conditions that interfere with trial observations and, in the physician’s judgment, the clinical trial should be terminated, the case will be treated as invalid.
(3) If a subject decides during the clinical trial that they no longer wish to participate and requests to withdraw from the study, their request should be respected, and they should be allowed to exit the trial.

### Allocation

This study will adopt a single-center randomized controlled trial design, randomly allocating 72 eligible patients into the acupuncture group or sham acupuncture group in a 1:1 ratio (36 patients in each group). A completely random digital sequence will be generated by third-party personnel using computer software, without setting any blocking or stratification factors, to ensure a fully randomized allocation process. After obtaining the patient’s informed consent form (**S2 File**), sequentially numbered, opaque, sealed, tamper-resistant, and water-resistant envelopes made of polypropylene (PP) will be utilized. The exterior of the envelopes will be labeled with only the random number and unsealing instructions, while group assignments will be concealed inside the envelopes to ensure allocation concealment. Recruitment personnel will screen and enroll patients who meet the inclusion criteria and distribute the envelopes in order. Before the intervention, the acupuncturist will open the envelope on-site to determine group assignment and administer the corresponding treatment.

### Blinding

In this trial, blinding will be implemented for participants, outcome assessors, and statistical analysts, with group assignments represented only as A or B. Statistical analysts will conduct the analysis without knowing the actual group assignments, and unblinding will occur only after the statistical analysis is completed. Due to the inherent characteristics of acupuncture, blinding will not be applied to acupuncture practitioners. The roles of acupuncture practitioners, outcome assessors, and statisticians will be carried out by separate individuals to ensure independence and minimize bias. To help maintain participant blinding, adhesive pads will be used in both groups to control for potential biases as much as possible. Unblinding will be permitted only in emergency situations. To assess the success of the blinding process, all participants will be asked to guess whether they received real or sham acupuncture within 5 minutes after their first treatment and again after their final treatment session.

### Interventions

In this trial, all patients will be advised to perform pelvic floor muscle training, following the exercise recommendations of K Bø and KEGEL [15,16]. During the baseline period, all patients will receive instructional guidance from professional rehabilitation physicians at the hospital. Subsequently, patients will be advised to perform pelvic floor muscle training daily, three times per day - morning, noon, and evening. The training program can be found in **S3 File**.

The acupuncture intervention will be conducted by two acupuncturists from the Beijing University of Chinese Medicine Third Affiliated Hospital, each with over 2 years of clinical experience. Patients will receive individual treatment to prevent communication about their treatments. During the treatment period, if a subject is in their menstrual cycle, treatment will be postponed until the end of the cycle. The delay time will not be included in the treatment period. During the trial, patients will not be allowed to use any specialized medications or rehabilitation treatments other than those specified in this research protocol, and any additional details will be reported in the Case Report Form (CRF). Within two weeks, patients will receive acupuncture and sham acupuncture treatments in a supine position, three times per week on alternate days. The specific procedures are as follows.

#### Acupuncture group.

**Fig 3:**
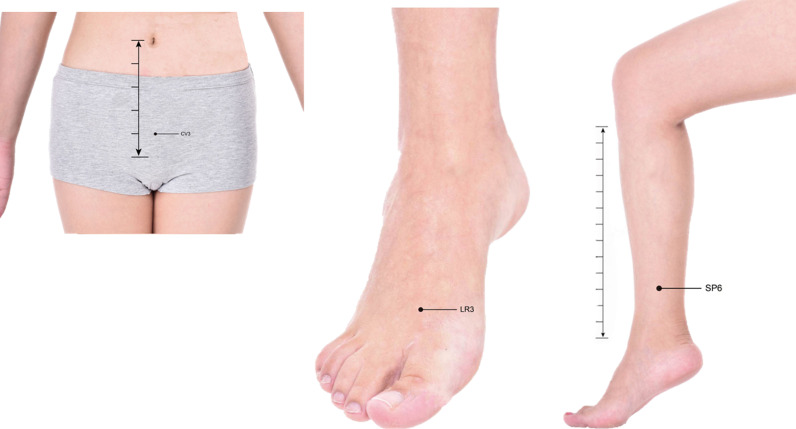
Acupoints. The original image is from the “Dajia Traditional Chinese Medicine App”.

The acupoints selected are bilateral Taichong (LR3), bilateral Sanyinjiao (SP6), and Zhongji (CV3), with the locations following the World Health Organization’s standard acupuncture positions. Patients will be placed in a supine position, and the acupoints will be sterilized with 75% alcohol. Disposable acupuncture needles will be used (diameter 0.30 mm, length 75 mm, Hwato brand, Suzhou Medical Appliance Factory, Ltd., China). Before needling, sterile adhesive pads will be placed on the acupoints. The acupuncture needles will penetrate through the sterile adhesive pads into the acupoints. The needles will be manipulated using lifting, thrusting, and rotational techniques to achieve ‘deqi’ sensation, and retained for 15 minutes. **Table 1** shows the acupoint locations, corresponding needle types, insertion angles, and insertion depths. Meanwhile, the specific acupoint locations are shown in **Fig 3**.

**Table 1:** Acupoints’ locations, corresponding needle type, Insert angle and depth of insertion.

Acupuncture name	Location	Needle type	Insert angle	Depth of insertion
Tai-chong (LR3)	On the dorsum of the foot, between the first and second metatarsal bones, in the depression distal to the junction of the bases of the two bones, over the dorsalis pedis artery.	0.30 × 75 mm	90°	10 ~ 20mm
San-yin-jiao (SP6)	On the tibial aspect of the leg, posterior to the medial border of the tibia, 3 cun superior to the prominence of the medial malleolus.	0.30 × 75 mm	90°	20 ~ 30mm
Zhong-ji (CV3)	On the lower abdomen, 4 cun inferior to the centre of the umbilicus, on the anterior median line.	0.30 × 75 mm	45°	30 ~ 40mm

1 cun (≈20 mm) is defined as the width of the patient’s thumb interphalangeal joint.

#### Sham acupuncture group.

The selected points are bilateral non-acupoint 1, non-acupoint 2, and unilateral non-acupoint 3. Patients will be placed in a supine position, and the non-acupoint locations will be sterilized with 75% alcohol. Disposable acupuncture needles will be used (diameter 0.30 mm, length 75 mm, Hwato brand, Suzhou Medical Appliance Factory, Ltd., China). Before needling, sterile adhesive pads will be placed on the non-acupoints. The acupuncture needles will penetrate through the sterile adhesive pads into the skin superficially, with insertion depth controlled within 2 millimeters, avoiding the ‘deqi’ sensation, and retained for 15 minutes. **Table 2** shows the non-acupoint locations, corresponding needle types, insertion angles, and insertion depths.

**Table 2:** Sham acupoints’ locations, corresponding needle type, Insert angle and depth of insertion.

Sham acupuncture name	Location	Needle type	Insert angle	Depth of insertion
Sham acupuncture 1 (SA1)	Between Taichong (LR3) and Gongsun (SP4), between the Liver Meridian and the Spleen Meridian	0.30 × 75 mm	90°	<2 mm
Sham acupuncture 2 (SA2)	1 cun lateral to Sanyinjiao (SP6)	0.30 × 75 mm	90°	<2 mm
Sham acupuncture 3 (SA3)	4 cun above the umbilicus, 1 cun to the left of the anterior midline, between the Kidney Meridian and the Stomach Meridian	0.30 × 75 mm	90°	<2 mm

1 cun (≈20 mm) is defined as the width of the patient’s thumb interphalangeal joint.

### Outcomes

#### Primary outcomes.

The primary outcome in this study is the change in the 1-hour pad test, which is a medical test recommended by the International Continence Society (ICS). The measurement method primarily involves instructing patients to complete a series of movements, and measuring the change in pad weight to determine the amount of urine leakage during the test process. The entire testing process is supervised by researchers, while the placement and removal of the pad is performed by the patients themselves. The 1-hour pad test can objectively and accurately measure the patient’s urine leakage volume and effectively observe changes in leakage volume after treatment, making it an objective and accurate indicator for observing therapeutic efficacy.

#### Secondary outcomes.

**72-hour bladder diary:** This refers to continuously recording (generally for 72 hours) without changing lifestyle or urination habits, documenting the type and volume of fluid intake, times of voluntary urination and volume per urination, as well as observing the severity of urinary incontinence, frequency of incontinence episodes, and pad usage over 72 hours. This can objectively reflect the patient’s urination and urinary incontinence conditions and serves as a means of treatment evaluation. The severity of PSUI will be graded based on urine leakage under normal conditions without strenuous activity (such as vigorous coughing, intense exercise, or heavy lifting) during the past 72 hours: none; mild, a few drops of leakage; moderate, soaking of underwear; severe, leakage soaking through outer clothing. For participants wearing pads, the severity of SUI will be graded as follows. Mild: a few drops of leakage; moderate: multiple leakages with patchy soaking of the pad; severe: single leakage with patchy soaking of the pad.

**ICIQ-SF questionnaire:** The International Consultation on Incontinence Questionnaire-Short Form. Used to investigate the degree of impact of urinary incontinence on patients, the brief questionnaire includes three scored items - frequency of urinary incontinence, amount of leakage, and impact on daily life - as well as a fourth non-scored item assessing the type of urinary incontinence. When filling out the form, patients are instructed to recall their symptoms. The total score of the three scored items constitutes the scale assessment result, with higher values indicating greater severity and lower values indicating less impact of urinary incontinence on the patient’s quality of life. This can relatively objectively quantify the impact on patients’ quality of life and psychological well-being, as well as the changes brought about by treatment.

**I-QOL questionnaire:** Incontinence Quality of Life questionnaire. Used to assess patients’ quality of life, including evaluation of three aspects: psychological impact, social barriers, and behavioral limitations caused by urinary incontinence. It consists of 22 questions, with each question having 5 levels, and each level scoring differently, ranging from 1 to 5 points. A lower total score indicates poorer quality of life due to urinary incontinence. This can relatively objectively quantify the impact on patients’ quality of life and psychological well-being, as well as the changes brought about by treatment.

**Pelvic floor muscle strength assessment:** A comprehensive evaluation is conducted using both instrument-based and manual methods. Instrument-based assessment is performed with the biofeedback rehabilitation device XCH-C1 (Shanghai Nuocheng Electric Co., Ltd.) to measure the strength of Type I and Type II muscle fibers in the pelvic floor before and after treatment. The results are graded on a scale of 0 to V, where 0 indicates no contraction and V indicates strong contraction.

Manual assessment follows the internationally standardized perineal muscle strength testing method, scoring pelvic floor muscle strength on a scale of 0–5 points. Higher scores correspond to stronger muscle strength. The manual grading system is as follows:

0: No contraction is felt.
1: Slight contraction is detected by the examiner’s fingers.
2: Muscle contraction is present, lasting for 2 seconds and repeated twice.
3: Contraction allows the examiner’s fingers to move upward and forward, lasting for 3 seconds and repeated three times.
4: Strong contraction resists finger pressure, lasting for 4 seconds and repeated four times.
5: Very strong contraction resists finger pressure, lasting for 5 seconds and repeated five times.

**Patient self-assessment of therapeutic efficacy:** Patients will conduct self-evaluations of treatment effectiveness at weeks 2, 4, and 6. A 4-point scale will be used to measure the level of help perceived by participants (0: no help; 1: little help; 2: moderate help; 3: significant help).

**Patient blinding assessment:** All patients will be asked to guess whether they received real or sham acupuncture within 5 minutes after their first treatment and after their final treatment.

**Assessment of other treatment methods:** Throughout the trial period, subjects will not be encouraged to undergo any specific PSUI treatments. For any treatments that have been used, relevant information should be recorded in the case report form. Other treatment methods for PSUI mainly include biofeedback electrical stimulation, vaginal dumbbells, and drug therapy.

### Safety evaluation and adverse events

Adverse reactions to acupuncture will be evaluated using an adverse reaction assessment form, which includes infections, hematomas, needle syncope, post-needling complications, and other adverse reactions. Throughout the trial, all Serious Adverse Events (SAEs) and Adverse Events (AEs) will be recorded and measured by both the subjects themselves and the acupuncturists. In our trial, SAEs will be defined as events requiring hospitalization, resulting in disability or impaired work capacity, life-threatening conditions, or death. AEs will be classified as treatment-related or non-treatment-related based on their potential correlation with the acupuncture procedure, as determined by the acupuncturist and relevant experts within 24 hours. Given that acupuncture is a minimally invasive treatment, pain that occurs within 30 minutes after needling and resolves spontaneously is not considered an AE due to the inevitable nature of needle-related discomfort. The number of AE cases and treatment sessions will be recorded. We will compare the proportion of subjects who experience treatment-related AEs. Subjects who experience at least one treatment-related adverse reaction will be included in the proportion comparison. If an adverse event is serious and related to this trial, the subject will withdraw from the study and receive appropriate medical care.

### Data collection and management

The screening phase will last for one week, which includes assessment of basic information such as age, gestational weeks, postpartum weeks, number of pregnancies, and delivery method, as well as detailed information about the subjects’ chief complaints, present illness history, past medical history, family history, and routine physical examination. According to the research protocol, we will collect and record the subjects’ original data completely and truthfully. Any data that significantly deviates from clinically acceptable ranges must be examined and explained. The completed and verified CRFs will be handed over to relevant personnel for data entry, management, and statistical analysis, and the data on the CRFs will not be modified. Any original data will be retained for 5 years after the completion of the study so that readers can access these data by contacting the corresponding author, except for patient identification information.

### Sample size

This study is a pilot trial, and for acupuncture clinical research, a sample size of 30 cases per group in pilot trials is suitable for parameter estimation [17], with a 20% dropout rate taken into consideration. Therefore, this study will enroll 36 subjects per group, for a total of 72 subjects.

### Statistical analysis

Statistical analysis will be performed using SPSS software version 26.0. Measurement data will be presented as means and standard deviations (SD) or medians and interquartile ranges (IQR), as appropriate. Counting data will be expressed as percentages.

Efficacy evaluation will adhere to the intention-to-treat (ITT) principle, including all patients who have received at least one treatment. For the primary outcome (1-hour pad test) and secondary outcomes (72-hour bladder diary, ICIQ-SF scores, I-QOL scores, pelvic floor muscle strength test results, and patient self-evaluation of efficacy), a mixed-effects model will be employed to account for the characteristics of repeated measures data. The model will include group, time points, and their interaction as fixed effects, with participants treated as random effects. Baseline values will be incorporated as covariates.

Missing data will be handled using multiple imputation. Five datasets will be generated based on age, sex, body mass index (BMI), baseline characteristics, and outcome values at other time points for analysis. Two sensitivity analyses will be performed for the primary outcome (1-hour pad test): (1) an analysis of the dataset without imputation to assess potential bias introduced by imputation and (2) an analysis of the per-protocol (PP) set to evaluate the impact of adherence. The PP set is defined as participants who completed at least 80% of the planned treatments without significant protocol violations (i.e., at least five treatments in both the acupuncture and sham acupuncture groups).

Safety analysis will include all participants who received at least one treatment. AE rates will be described using counts and percentages. Between-group comparisons will be conducted using the chi-square test when the expected frequency requirements are met; otherwise, Fisher’s exact test will be used. All statistical analyses will be two-tailed, with a significance level of *P *< 0.05 considered statistically significant.

### Exploratory analysis

Exploratory analysis will be conducted to explore potential correlations between treatment outcomes and baseline characteristics. Multiple linear regression models will be used, with key outcomes (e.g., change in 1-hour pad test results, ICIQ-SF scores, and I-QOL scores) as dependent variables. Independent variables will include baseline characteristics such as age, BMI, pelvic floor muscle strength, and other relevant factors. Results will be presented as regression coefficients with 95% confidence intervals. This analysis aims to identify predictors of treatment response and potential moderating factors.

### Study status

This trial is currently recruiting subjects. After trial registration, the first patient was recruited on September 1, 2024. Recruitment is expected to be completed by June 2025. Data collection will be completed in July 2025, and the test results are expected to be obtained in September 2025.

## Discussion

This study represents the first randomized controlled trial of acupuncture treatment for PSUI using sham acupuncture as a control. Previous acupuncture studies for PSUI have typically lacked appropriate placebo controls, making it difficult to distinguish between the specific therapeutic effects of acupuncture and non-specific effects [18–20]. By employing sham acupuncture as a control, combined with a rigorous blinding design, this study aims to provide more reliable evidence for the specific therapeutic efficacy of acupuncture in treating PSUI.

In terms of methodological design, several measures are implemented to ensure research quality: participant blinding is maintained through the use of adhesive pads to conceal both real and sham acupuncture applications; independent outcome assessors and statistical analysts are employed to reduce measurement bias; software-generated random number sequences and opaque sealed envelopes are utilized to ensure effective implementation of randomization and allocation concealment.

Regarding outcome measures, this study employs the 1-hour pad test as the primary outcome, providing an objective measure of therapeutic efficacy. Secondary outcomes include 72-hour bladder diary, ICIQ-SF, and I-QOL, etc. The combination of objective indicators with subjective reports is used to comprehensively evaluate treatment effectiveness. Compared to existing biofeedback and electrical stimulation therapies, acupuncture offers advantages of simpler operation and relatively lower costs. The treatment frequency (three times per week) and duration (two weeks) established in this study are reasonably designed to promote patient compliance. Current research indicates that acupuncture can improve pelvic floor function and enhance urethral sphincter strength [9,11,21].

The acupoint combination selected in this study has substantial theoretical foundation: Taichong (LR3) can enhance control over micturition reflex [22,23], Zhongji (CV3) has bidirectional regulatory effects on bladder tension [24–26], while Sanyinjiao (SP6) can improve bladder and urethral control of urination through regulation of the central nervous system [27]. This acupoint combination, based on Traditional Chinese Medicine theory, may produce synergistic effects, thereby achieving better therapeutic outcomes.

However, this study has the following limitations: First, as a pilot trial, the sample size of 36 participants per group may be insufficient to draw definitive conclusions. Second, considering the use of sham acupuncture in the control group, the duration of non-essential interventions was minimized to adhere to ethical guidelines. Additionally, as the majority of participants were lactating women, the 2-week intervention period was designed to reduce time burden and potential interference with breastfeeding caused by prolonged procedural complexity. Furthermore, the short 4-week follow-up period may limit the evaluation of long-term efficacy. Finally, the single-center design could affect the external validity of the results. Nevertheless, as this is an exploratory pilot trial, our findings will inform critical parameters—such as the necessity to extend intervention and follow-up durations—for future large-scale follow-up studies.

Regarding safety aspects, this study establishes a comprehensive adverse event monitoring and reporting system, clearly distinguishing between treatment-related and non-treatment-related adverse events, and sets specific safety evaluation criteria. Given the minimally invasive nature of acupuncture treatment, anticipated adverse reactions are primarily limited to mild needling sensation or local discomfort, which are typically transient and self-resolving.

Postpartum stress urinary incontinence significantly impacts women’s quality of life and psychological well-being. While current conservative treatments such as PFMT are effective, they require extended training periods and suffer from poor patient compliance [28]. Although biofeedback and electrical stimulation therapies show certain therapeutic effects, they are costly and not covered by medical insurance. In this context, acupuncture therapy holds significant importance as an economical and safe alternative or complementary treatment option. Therefore, this study has important clinical translational value.

Through standardized treatment protocols and rigorous methodological design, this study not only provides reference for clinical practice but also lays the foundation for subsequent large-scale clinical research. Based on the results of this pilot study, future research will explore the following directions: (1) expanding sample size for confirmatory studies; (2) extending follow-up periods to evaluate long-term efficacy; (3) conducting multi-center studies to enhance result generalizability; (4) exploring optimized protocols combining acupuncture with other treatment modalities; (5) further investigating the mechanisms of action in acupuncture treatment for PSUI; (6) exploring factors influencing treatment effectiveness. This pilot study, employing rigorous methodological design, will provide preliminary evidence-based medical evidence for acupuncture treatment of PSUI, carrying significant clinical and social value.

## Supporting information

S1 File SPIRIT checklist. (DOCX)

S2 File Informed consent form. (DOCX)

S3 File Pelvic floor muscle training program. (DOCX)
