# Supplementary material for: Efficacy and safety of acupuncture for early postpartum stress urinary incontinence: A protocol for a pilot randomized controlled trial
Source: PLoS One. 2025 May 27;20(5):e0324384. doi: 10.1371/journal.pone.0324384 (PMC12112143; doi:10.1371/journal.pone.0324384)
Supplement: S2 File — (DOCX) [file pone.0324384.s002.docx]

**Informed Consent Form • Information Sheet**

**Dear Madam,**

Your doctor has confirmed that you are experiencing postpartum stress urinary incontinence. We invite you to participate in the "Randomized Controlled Pilot Study on Acupuncture Treatment for Early Postpartum Stress Urinary Incontinence." This project is funded independently by the Beijing University of Chinese Medicine Third Affiliated Hospital. The study plans to recruit 72 voluntary participants, with the research scheduled to commence in September 2024. This project has been reviewed and approved by the Research Ethics Committee of the Beijing University of Chinese Medicine Third Affiliated Hospital.

Before deciding whether to participate in this study, please read the following information carefully. It will help you understand the purpose of the study, the procedures, and the timeline, as well as the potential benefits, risks, and discomforts associated with participation. If you wish, you may discuss this with your family or friends or consult your doctor for clarification to assist in your decision-making until you fully understand this clinical trial.

Your participation in this study is voluntary. If you agree to participate, please confirm your consent by signing the statement on the informed consent form after thoroughly reviewing the provided materials.

**1. Research Background and Objectives**

Postpartum stress urinary incontinence (SUI) is a common condition experienced by women after pregnancy and childbirth, with an incidence rate ranging from 9% to 45%. In China, the incidence of stress urinary incontinence within 42 days postpartum among first-time mothers is 13.86%, rising to 18.93% at three months postpartum. The functional changes in the pelvic floor structure caused by childbirth are temporary, and the period between 42 days and three months postpartum is considered the golden window for pelvic floor rehabilitation. The first year postpartum represents the optimal time for treatment and prevention. Taking advantage of this critical one-year period and identifying effective treatment methods are key to recovery.

This study aims to further explore the role of acupuncture in the treatment of postpartum stress urinary incontinence. The goal is to comprehensively and objectively evaluate the efficacy and safety of acupuncture therapy in patients with postpartum SUI. By doing so, the study seeks to provide patients with more treatment options and effective therapeutic approaches. Furthermore, the findings will contribute new directions for the clinical treatment of postpartum stress urinary incontinence while offering strong support for the application of traditional Chinese medicine in modern medical practice.

**2. Primary Target Population of This Study**

**2.1 Diagnostic Criteria:**

(1) Based on the joint report by the International Continence Society (ICS) and the International Urogynecological Association (IUGA) on the terminology for assessing and managing obstetric pelvic floor disorders: Complaints of involuntary leakage of urine during effort, physical exertion (including physical activities), sneezing, or coughing within 12 months

(2) According to the Guidelines for the Diagnosis and Treatment of Female Stress Urinary Incontinence (2017 edition): a. Symptom-based diagnosis: Uncontrollable urine leakage from the urethra occurs during sudden increases in abdominal pressure, such as jumping, lifting heavy objects, coughing, or sneezing, and stops when abdominal pressure is relieved; b. Sign-based diagnosis: Positive 1-hour pad test or stress-induced urinary leakage during provocation testing confirms leakage under increased pressure conditions; c. Exclusion of other types of SUI or bladder diseases. The above criteria collectively confirm the diagnosis.

(3) Subjective grading is based on the Ingelman-Sundberg classification: a. Mild: Leakage occurs during coughing or sneezing, and pads are not required; b. Moderate: Leakage occurs during activities such as running, jumping, or brisk walking, and pads are required; c. Severe: Leakage occurs during minimal activities or while lying down or changing positions.

(4) Objective grading is determined by the weight difference in the pad test: a. Dry:≤1g; b. Mild leakage: 1.1–9.9g; c. Moderate leakage:10–49.9g; d. Severe leakage:≥50g. Note: A weight difference of 1 gram may be due to weighing errors, sweating, or vaginal secretions.

**2.2 Inclusion Criteria:**

(1) Age 20–40 years.

(2) Between 42 days and 1 year postpartum.

(3) Experience urine leakage during coughing, sneezing, laughing, or physical activity, stopping after the pressure ends, occurring at least once in three months.

(4) Weight increase of >1g and <50g in the 1-hour pad test.

(5) Classified as mild or moderate SUI according to the Ingelman-Sundberg classification.

(6) Willingness to participate in the study and signing of the informed consent form.

Any "No" for the above criteria disqualifies participation in the trial.

**3. Individuals Unsuitable for Participation**

(1) Multiple pregnancies.

(2) Symptoms of urinary incontinence prior to pregnancy.

(3) Symptoms of urinary frequency or urgency.

(4) Symptoms of urinary tract infection.

(5) Postpartum lochia not resolved or uterine cavity retention after delivery.

(6) Co-existing uterine or other gynecological diseases.

(7) History of severe complications such as macrosomia, prolonged second stage of labor, placenta previa, threatened preterm labor, or pregnancy-induced hypertension.

(8) History of cesarean section indications or contraindications to vaginal delivery.

(9) Previous surgery for urinary incontinence or pelvic floor surgery.

(10) Pelvic organ prolapse greater than grade 2.

(11) Overactive bladder syndrome or urological tumors.

(12) Weak detrusor muscle contraction.

(13) Receipt of continuous or specific treatment for SUI or medication affecting bladder function within the past month.

(14) Severe cardiovascular, cerebral, hepatic, renal, or psychiatric diseases, diabetes, multiple system atrophy, cauda equina syndrome, or spinal cord injury.

(15) Presence of a cardiac pacemaker, metal allergies, or severe fear of needles.

(16) Participation in another clinical study within the past 3 months.

Any "Yes" for the above criteria disqualifies participation in the trial.

**4. What You Will Need to Do if You Participate in the Study**

(1) Before you are enrolled in the study, you will undergo the following checks to determine your eligibility:

You will be required to provide a medical history, undergo a physical examination, a routine urine test, a 1-hour pad test, and a pelvic floor muscle strength assessment. You will be asked to fill out a 72-hour bladder diary, ICI-Q-SF questionnaire, and a urinary incontinence quality of life form based on your actual situation to determine your eligibility for the study.

Among the treatments or checks mentioned above, sham acupuncture is a study-specific treatment (i.e., if you do not participate in this study, you would not need to undergo this treatment).

(2) If you pass the above checks, the study will proceed as follows:

This study is a randomized controlled clinical trial with a total duration of 6 weeks, including a 2-week treatment period and a 4-week follow-up period. If you are willing to participate in this study, you will receive professional guidance from a rehabilitation physician until you master the pelvic floor muscle training (PFMT) method. For the next 2 weeks, you will need to conduct home training on your own, three times a day (morning, noon, and evening). You will have a 50% chance of receiving traditional Chinese acupuncture treatment, which includes three sessions per week, each lasting 15 minutes, for a total of 6 sessions over 2 weeks. You will also have a 50% chance of receiving traditional Chinese sham acupuncture treatment, which includes the same schedule and duration as the acupuncture treatment. After the treatment, we will conduct a 4-week follow-up, during which you will be required to fill out a 72-hour bladder diary, ICI-Q-SF questionnaire, and a urinary incontinence quality of life form each week, and self-assess the treatment effect. At the end of the treatment and the follow-up period, you will undergo a 1-hour pad test and a pelvic floor muscle strength assessment again.

(3) Important reminders for participants:

During the study, you must cooperate with the doctors to complete the acupuncture treatment and related checks. You are prohibited from participating in other clinical trials at the same time. If you need to use other medications or treatment methods due to special circumstances, you must contact the doctor and fill out the CRF form in a timely manner.

**5. Possible Risks and Discomforts of Participating in the Study**

If you experience any discomfort during the study, or if your condition changes or any unexpected situation arises, regardless of whether it is related to the medication, please notify your doctor immediately. He or she will make a timely judgment and provide medical treatment. (Study intervention) There may be cases where the treatment is ineffective, and the condition may continue to progress due to ineffective treatment or the presence of other diseases. All acupuncture treatments may have adverse reactions. While observing the therapeutic effects, it is important to monitor for adverse reactions, such as pain, bleeding, hematoma, acupuncture fainting, or infection at the acupuncture site during the process. If acupuncture fainting is observed, stop the needling or remove the regular filiform needles immediately and manage symptomatically. Generally, symptoms can be relieved after rest; if pain, bleeding, or hematoma at the needle site is found, disinfect and manage promptly, and hematomas usually resolve on their own. The filiform needle acupuncture used in this study is a routine clinical treatment for postpartum diseases, and even if you do not participate in this clinical study, as long as you receive this treatment method, these adverse reactions may occur. In the event of acupuncture complications and adverse reactions, an adverse reaction management protocol will be initiated.

Any treatment may be ineffective, and there may be evidence suggesting that the acupuncture treatment measures in this study have satisfactory efficacy, but this does not guarantee that it will definitely be effective for you. The acupuncture treatment measures used in this study are not the only treatment for postpartum stress urinary incontinence. If the treatment is ineffective for your condition, you can inquire with your doctor about possible alternative treatment methods. Additionally, the condition may continue to progress due to ineffective treatment or the presence of other diseases.

If you experience any discomfort or condition changes during the study, please contact your doctor promptly, and we will provide timely treatment or management. If the researchers receive information that may affect your continued participation in the trial, they will inform you in a timely manner.

**6.Acupuncture Complications and Adverse Reaction Management Plan**

(1) Acupuncture Fainting (Needle Fainting)

Symptoms: The patient may experience pale complexion, sweating, dizziness, palpitations, and in severe cases, syncope.

Management:

a. Immediately stop acupuncture and remove the needles when the patient experiences needle fainting.

b. Have the patient lie down with their head slightly lower and ensure good air circulation. Provide warm water or sugar water and gently pat the patient's cheeks.

c. If symptoms do not subside, further examinations will be conducted, such as measuring blood pressure and blood sugar levels.

d. Intravenous fluid administration may be necessary to replenish electrolytes and improve circulation.

e. Electrocardiogram (ECG) monitoring will be performed to closely observe vital signs and prevent recurrent syncope.

(2) Needle Retention (Needle Stuck)

Symptoms: The needle cannot be removed after acupuncture, and the patient feels pain or discomfort.

Management:

a. Comfort the patient and guide them to relax. Gently press around the acupuncture point with another finger, slowly rotate or lift the needle, and gradually remove it.

b. If the needle still cannot be removed, seek assistance from an experienced acupuncturist promptly.

c. If the needle is difficult to remove, an X-ray or ultrasound examination will be conducted to determine the position and depth of the needle.

d. Under anesthesia, the physician will perform a small incision surgery to remove the retained needle.

e. Postoperative disinfection will be carried out, and antibiotics may be administered to prevent infection.

(3) Needle Breakage

Symptoms: The needle breaks and remains under the skin.

Management:

a. Keep the patient calm and minimize their movement.

b. Immediately remove the visible part of the needle.

c. If the needle is completely under the skin, mark the location.

d. Imaging localization: Accurately locate the position of the broken needle using X-ray, CT, or MRI.

e. Surgical needle removal: Perform surgery under local or general anesthesia to cut open the skin and soft tissue and remove the broken needle.

f. Postoperative care: Prevent infection and promote wound healing, which may require antibiotic treatment and regular dressing changes.

(4) Visceral Injury

Symptoms: Severe pain, bleeding, difficulty breathing, and a drop in blood pressure after acupuncture.

Management:

a. Immediately stop acupuncture and remove the needles. Have the patient lie down and remain still.

b. Emergency treatment: Depending on the situation, emergency surgery may be required. For example, if the lung is punctured, a chest closed drainage procedure may be necessary; if the abdomen is punctured, a laparotomy may be needed to inspect and repair internal organ damage.

c. Intensive care: Postoperatively, the patient may be admitted to the Intensive Care Unit (ICU) for close monitoring of vital signs and supportive treatment.

d. Preventing complications: Postoperative antibiotic administration to prevent infection, nutritional support, and other symptomatic treatments.

(5) Infection

Symptoms: Redness, pain, and pus formation at the acupuncture site.

Management:

a. Local treatment: For mild infections, perform local debridement, disinfection, and apply antibiotic ointment.

b. Systemic antibiotic therapy: If the infection is severe, oral or intravenous antibiotics may be required.

c. Wound management: For purulent or severe infections, drainage and dressing changes may be necessary to promote healing.

All treatment costs resulting from complications and adverse reactions that may occur due to acupuncture operations in this study will be borne by our research team.

**7. Alternative Diagnostic and Treatment Methods Outside of This Study**

You have the option not to participate in this study or to withdraw from it at any time. There are various alternative treatment methods available, such as moxibustion and traditional Chinese medicine, and you are not obligated to choose to participate in this study for the treatment of your condition. If necessary, your physician will provide helpful reference opinions, any required and useful treatments, or refer you to other departments for consultation. This will not have any adverse effects on your access to routine medical care.

**8. Potential Benefits of Participating in the Study**

By participating in this study, you will have the opportunity to receive acupuncture treatment at no cost, which may potentially improve your condition to some extent. During the study period, you will also receive high-quality medical services.

We cannot guarantee an improvement in your health status, but we hope that the information obtained from your participation in this study will be beneficial for other patients with similar conditions in the future.

Signing this informed consent form does not deprive you of any legal rights or interests. Your decision to participate in this study is entirely voluntary, and you have the right to withdraw from the study at any time without any impact on your medical care or legal rights.

**9. Costs Associated with the Study**

The Beijing University of Chinese Medicine Third Affiliated Hospital will cover the costs of examinations and medical services related to your participation in this study, including the acupuncture treatment provided as part of the study.

If you require treatments and examinations for other concurrent diseases, these will not be covered by the sponsor.

The doctors and the research team of the " Randomized Controlled Pilot Study on Acupuncture Treatment for Early Postpartum Stress Urinary Incontinence " will make every effort to prevent and treat any harm that may arise from this study. In the event of any trial-related injury, the sponsor will cover your medical expenses and provide appropriate financial compensation, as stipulated in China's "Good Clinical Practice for Drug Clinical Trials."

**10. Confidentiality of the Study**

All information about you, including your identity, medical history, condition, physical examinations, and laboratory test results, will be strictly confidential within the limits allowed by law. Researchers, representatives of the sponsor, the ethics committee, and the drug regulatory authorities will be allowed to review your medical records related to this study to verify the authenticity and accuracy of the data collected, but they will not have access to your personal details. Any public materials or reports on the results of this study will not disclose your personal identity.

**11. Grievances and Complaints from Participants**

If you have any medical issues related to this study, please contact Dr. Xu at the phone number: 18810383185.

Should there be any significant new information during the study that may affect your willingness to continue participating, your doctor will notify you in a timely manner.

If you wish to express any dissatisfaction or concerns about participating in this study, or if your personal rights are infringed upon, you may contact the Research Ethics Committee of the Beijing University of Chinese Medicine Third Affiliated Hospital at the phone number: 010-84980751.

**12. Your Rights and Interests**

Participation in the study is entirely voluntary. You have the right to refuse to participate in this study, or to withdraw from the study at any time during the process, without affecting your relationship with your doctor or causing any loss to your medical care or other interests.

If there are circumstances during the study that make it inappropriate for you to continue participating, your doctor or researcher may suggest that you withdraw or discontinue participation at any time for your best interests.

If you choose not to participate in this study or decide to withdraw at any point, there are many other alternative treatments available, such as medication, moxibustion therapy, traditional Chinese medicine, and prepared traditional Chinese medicine treatments. You are not required to participate in this study for the treatment of your condition.

If you withdraw from the study for any reason, you may be asked about your acupuncture treatment experience. If deemed necessary by your doctor, you may also be requested to undergo laboratory tests and physical examinations. This is beneficial for the protection of your health.

**Informed Consent Form • Consent Signature Page**

**Participant's Statement**

I have carefully read the information provided for the clinical study of " Randomized Controlled Pilot Study on Acupuncture Treatment for Early Postpartum Stress Urinary Incontinence " and fully understand the purpose, content, methods, as well as the potential benefits and risks of participating in this clinical study. The medical terms have been clearly explained by the doctor, and all my questions have been answered in a way that is easy to understand. I understand that I can refuse to join the study or withdraw from it at any time and under any circumstances without discrimination or retaliation, and my medical treatment and rights will not be affected.

My participation in this study is entirely voluntary, and I have had ample time to consider it thoroughly. I am aware of the therapeutic effects and potential risks that the study's medical devices may bring to my condition. I have received complete and accurate information related to this study, and I fully understand and support this clinical research. I ensure that I have provided the researchers with an honest account of my medical history, medication history, and participation in clinical trials. I have decided to participate in this clinical study voluntarily, without any pressure, and with the freedom to choose, and I will endeavor to follow the requirements of the protocol and the suggestions of the researchers.

I agree to allow the drug regulatory authorities, clinical research auditors and monitors, the ethics committee, or representatives of the sponsor to review my research materials.

I will receive a copy of the signed and dated informed consent form.

(Please sign in black ink with regular script, and signatures cannot be replaced by a stamp)

**Participant's Signature: Date:**

**Contact Phone Number (Mobile):**

Note: If the participant is unable to sign the informed consent form due to lack of capacity or other reasons, it should be signed by their legal guardian.

**Guardian's Signature: Date:**

**Relationship to the Participant:**

**Contact Phone Number:**

**Reason for the Participant's Inability to Sign:**

If the participant or their legal guardian lacks the ability to read, a witness should be present during the informed consent process. After a thorough explanation of the consent information, the witness will read the informed consent form document to ensure it aligns with the oral information provided. The participant or their legal guardian will then give their oral consent, and the witness will sign the informed consent form document and note the date.

I confirm that the information in the informed consent form has been correctly interpreted and that the participant and/or the participant's legal representative has understood this information. The participant voluntarily consents to participate in this study.

**Fair Witness Signature: Date:**

**Contact Phone Number:**

**Investigator's Statement**

I confirm that I have explained the details of this trial to the subject, including her rights as well as the potential benefits and risks, and have given her ample time to read the informed consent form, discuss with others, and have her questions about the study answered; I have informed the patient of the contact information in case of problems; I have informed the patient that she can withdraw from the study at any time during the study period without any reason.

**Investigator Signature: Date:**

**Contact Phone Number (Landline):**

**Contact Phone Number (Mobile):**
