# Supplementary material for: Efficacy and safety of acupuncture for early postpartum stress urinary incontinence: A protocol for a pilot randomized controlled trial
Source: PLoS One. 2025 May 27;20(5):e0324384. doi: 10.1371/journal.pone.0324384 (PMC12112143; doi:10.1371/journal.pone.0324384)
Supplement: S3 File — (DOCX) [file pone.0324384.s003.docx]

**Pelvic Floor Muscle Training Program**

Before the first training session, provide the patient with relevant anatomical and physiological knowledge, as well as information about the methods and precautions of pelvic floor training. A simple approach can be asking the patient to perform actions such as interrupting the urination process or inhibiting the anal gas expulsion process to help them perceive the correct muscle contractions.

1. Teaching Guidance: The physician provides one-on-one instruction to the patient, guiding them through training in supine, standing, and sitting positions. Before training, instruct the patient to empty their bladder. In each position, the patient should spread their legs apart, while relaxing other pelvic muscles, to focus on strengthening the pelvic floor muscles. The patient is instructed to contract the pelvic floor muscles with maximum effort for 8 seconds, followed by 8 seconds of relaxation, and then perform 4 rapid contractions lasting 1-2 seconds each. Repeat this sequence 12 times to complete one set of training. After changing positions, repeat the same training. In each position, the patient should perform at least 12 sustained pelvic floor contractions with maximum effort. The physician will guide the patient until they have fully mastered the technique and can independently perform home exercises.

2. Home Training: Every day, the WeChat mini-program will send exercise reminders and follow-along videos to the participants via WeChat, in the morning, noon, and evening. The patient can choose their preferred position for training. The training content remains the same as before, with one set of exercises each time (including 12 sustained contractions and 48 rapid contractions). The patient should train three times a day—morning, noon, and evening—continuing the training for 2 weeks. It is recommended that patients perform the exercises before or after activities they regularly do, based on their daily routine. Different positions can be used, such as before getting out of bed in the morning (supine), after lunch (sitting), or while watching TV after dinner (standing).

3. Precautions for Pelvic Floor Muscle Training: Proper contraction of the pelvic floor muscles is more important than forceful contractions. Patients must correctly identify the pelvic floor muscles that need to be trained. Pelvic floor muscle training focuses on strengthening the muscles around the vagina and anus. While contracting the pelvic floor muscles, patients should make every effort to avoid contracting the abdominal, back, and thigh muscles.
